# Iodine Fortification of Edible Legume Sprouts: A Pilot Biofortification Study

**DOI:** 10.3390/foods14213691

**Published:** 2025-10-29

**Authors:** Paweł Paśko, Ewelina Prochownik, Jadwiga Kryczyk-Kozioł, Molka Jlassi, Dhouha Yahyaoui, Agnieszka Galanty, Hela Ben Ahmed, Justyna Dobrowolska-Iwanek

**Affiliations:** 1 Department of Food Chemistry and Nutrition, Faculty of Pharmacy, Jagiellonian University Medical College, 30-688 Kraków, Poland; p.pasko@uj.edu.pl (P.P.); ewelina.gajdzik@uj.edu.pl (E.P.); jadwiga.kryczyk@uj.edu.pl (J.K.-K.); 2 Laboratory of Interactions Plant-Soil and Environment, Faculty of Science, Tunis El Manar University, Tunis 2092, Tunisia; molka.jlassi@etudiant-fst.utm.tn (M.J.); dhouha.yahyaoui@etudiant-fst.utm.tn (D.Y.); hela.benahmed@fst.utm.tn (H.B.A.); 3 Department of Pharmacognosy, Faculty of Pharmacy, Jagiellonian University Medical College, 30-688 Kraków, Poland; agnieszka.galanty@uj.edu.pl; 4 Mixed Tunisian Moroccan Laboratory of Plant Physiology and Biotechnology and Climate Change, Tunis El Manar University, Tunis 2092, Tunisia

**Keywords:** iodine, sprouts, fortification, legumes

## Abstract

Iodine has an essential role in the human body; however, its insufficiency is still a challenge. Therefore, the search for new strategies to increase iodine intake in the daily diet is fully justified, with sprouts as a preferred and interesting candidates for biofortification. The aim of this pilot study was to investigate the effect of different methods of iodine enrichment of legume sprouts (yellow lupine, lentil, red and white clover, and common vetch) as well as to identify the most promising species for iodine bioaccumulation. The iodine content in mineralized sprout extracts was determined using the Sandell–Kolthoff method. Watering seeds, previously soaked in water (1 day), with a 6.5 mg/L potassium iodide solution (7 days) revealed to be the most effective fortification model, achieving the highest iodine concentrations in all tested species, with white clover being the best accumulator (1026.7 ± 60.4 µg I/100 g fresh weight) of this component. In turn, the greatest changes in biomass were observed in red clover (even up to 250% of the control). Iodine biofortification of legume sprouts could be effective; nevertheless, further research in this area is required.

## 1. Introduction

Iodine is an essential micronutrient crucial for human health, primarily due to its role in the synthesis of thyroid hormones, which regulate metabolism, growth, and development. Despite its importance, iodine deficiency remains a widespread global health issue, particularly in regions with iodine-depleted soils. Additionally, our recent research suggests that young people, especially those following vegan and vegetarian diets, are increasingly at risk of iodine deficiency. This is largely due to the elimination of primary dietary sources of iodine, such as dairy products, salt and fishes [1]. Addressing this concern, a number of iodine fortification strategies have been developed as a dietary intervention support. These include, apart from iodized salt introduction, the development of biofortification strategies—enriching crops with essential nutrients such as iodine—as a promising approach to combat micronutrient deficiencies and enhance food security. However, despite iodized salt and the widespread introduction of other fortified products, the prevalence of thyroid disorders linked to iodine deficiency continues to rise globally. This is particularly concerning for individuals adhering to plant-based diets, which may inherently lack adequate iodine and thereby increase the risk of thyroid dysfunction. On the other hand, it is also important to be aware of the risks associated with the excessive iodine intake, including hyperthyroidism, thyroid autoimmunity, or thyroid cancer [2].

Sprouts have emerged as an attractive food candidate for biofortification due to their visual appeal, low caloric content, and popularity among health-conscious consumers. Sprouts are not only rich in macronutrients and essential trace elements but also provide interesting textures and are easy to incorporate into modern diets [3]. Moreover, sprouts, as rapidly growing plant systems with high metabolic activity, are particularly well-suited for biofortification. Their ability to efficiently absorb and incorporate nutrients makes them excellent carriers for iodine delivery in the human diet. Iodine biofortification of the sprouts can thus increase their nutritional value by boosting iodine content and potentially enhancing other beneficial properties, such as antioxidant capacity and growth performance [4]. Various chemical forms of iodine, such as iodide (I^−^) and iodate (IO_3_^−^), have been utilized in plant biofortification, each interacting differently with plant physiology. Iodate has been shown to enhance plant growth and metabolic activity without causing toxicity, while excessive iodide can sometimes result in phytotoxic effects [5]. This underscores the importance of optimizing both the chemical form and concentration of iodine used in biofortification. Beyond improving human nutrition, iodine biofortification may confer agronomic benefits, including improved plant growth, enhanced resistance to abiotic stress, and increased metabolic efficiency [6]. These dual benefits make iodine biofortification a promising intervention from both food science/agricultural and public health perspectives.

In our earlier analyses, the sprouts from eight botanical families, including several representatives of the Fabaceae family, were investigated, in terms of their content of micro and macronutrients. Interestingly, considerable variability was observed in the iodine content among these legumes. Mung bean sprouts displayed the highest average iodine concentration, reaching 0.43 mg/100 g of dry matter, whereas kidney bean and pea sprouts exhibited the lowest values, both approximately 0.03 mg/100 g of dry matter [3]. These differences pointed out that legume sprouts are a valuable model for further in-depth studies on micronutrient profiles, especially as they are also rich in bioactive phytochemicals—such as flavonoids and isoflavones—known for their chemopreventive potential [7]. Isoflavones can reveal estrogenic effect, due to their unique chemical structure, resembling human estrogens. This may translate to their effect on the viability and proliferation of estrogen-sensitive human cancer cells, like breast, prostate or thyroid, through both ER-dependent and ER-independent pathways [8]. Especially, the ability of isoflavones to inhibit this process in thyroid cells, including thyroid malignancies, remains an open and promising area of research [9]. Thus, legume sprouts, with their potential influence on thyroid function, are particularly relevant in terms of iodine fortification, especially since this aspect is so far a novelty for this group of plants.

In our preliminary screening, a broad panel of legume seeds, including peas, chickpeas, and various beans, was initially tested. However, under the applied experimental conditions, most of these species exhibited poor germination efficiency or were highly susceptible to decay during iodination process. Therefore, five legume species—yellow lupine, lentil, red and white clover, and common vetch—were selected for further study. These species demonstrated good stability during the treatment process and effective iodine accumulation. Moreover, some of them, such as lentil and clover, are already known as edible or forage crops with nutritional potential, while yellow lupine and common vetch represent promising candidates for the development of novel functional or alternative protein sources.

Focusing on iodine biofortification of plants—mainly sprouts—offers a sustainable, cost-effective, and environmentally friendly approach to reducing iodine deficiency while supporting human health. Sprouts are especially suitable for this application, as the leaves are the second most efficient organ (after roots) for iodine accumulation. Therefore, the aim of this study was to explore for the first time how different iodine fortification methods affect the concentration of this trace element in legume sprouts. The goal was to develop the optimal fortification treatment, and identify the most promising species for iodine accumulation but also evaluate their impact on biomass and overall plant performance, providing a novel perspective on enhancing their nutritional profile.

## 2. Materials and Methods

### 2.1. Chemicals

Ultrapure water of 18 MΩ cm was obtained from Milli-Q Direct-Q^®^ 3 UV Water Purification (Merck-Millipore, Burlington, MA, USA). ReagentPlus^®^ arsenic (III) oxide, and ammonium cerium (IV) sulfate dehydrate (ACS grade) were from Sigma-Aldrich (Seelze, Germany), 98% sulfuric acid and ammonium peroxodisulfate (ACS grade) were from Supelco (Darmstadt, Germany). Sodium chloride was obtained from Chempur (Piekary Śląskie, Poland). The 0.1 M arsenious acid solution was prepared following the procedure described by Machado et al. [10]. Briefly, 5 g of arsenic (III) oxide and 12.5 g of sodium chloride were dissolved in 200 mL of 2.5 M sulfuric acid. Subsequently, 250 mL of water was added, and the mixture was brought to a boil. After cooling to room temperature, the solution was adjusted to a final volume of 500 mL, yielding final concentrations of 100 mM arsenious acid, 0.43 M NaCl, and 1 M sulfuric acid.

### 2.2. Seeds for Sprouts

The seeds used for sprouting in this study included five species from the *Fabaceae* family. Yellow lupine (*Lupinus luteus* L.) seeds were sourced from AGRONAS Sp. z o.o. (Koło, Poland), with reference number PL030/09/10060/Z276/A. Lentil (*Lens culinaris* Medik.) seeds were obtained from Bavicchi, distributed in Poland, reference number 8003419028629. Red clover (*Trifolium pratense* L.) seeds were purchased from TORAF Sp. z o.o. (Kujakowice Górne, Poland), reference number 82595CIN0DS. White clover (*Trifolium repens* L.) seeds were supplied by Małopolska Hodowla Roślin Sp. z o.o. (Kraków, Poland), under reference number 201654674. Finally, common vetch (*Vicia sativa* L.) seeds were also obtained from Małopolska Hodowla Roślin Sp. z o.o. (Kraków, Poland) with the reference number PL012/61/5536/Z254/A. All voucher specimens were deposited in Department of Food Chemistry and Nutrition, Jagiellonian University, Kraków, Poland. In the whole manuscripts these five species were denoted as follows: yellow lupine (YL), lentil (L), red clover (RC), white clover (WC), and common vetch (CV).

### 2.3. Sprouting Conditions and Harvesting Procedure

Before initiating the experiments, all seed batches were standardized to ensure uniform initial conditions. Only seeds of similar size and plumpness were selected, excluding damaged or shriveled ones. Seeds were derived from the same harvest year and stored for less than 12 months under controlled conditions (dark, dry, and cool environment at 18 ± 2 °C, relative humidity below 40%). Prior to the cultivation, seed viability was verified through a germination rate screening on moistened filter paper (100 seeds per species, three replicates). Only batches showing ≥ 90% germination rate were used for biofortification experiments.

**Figure 1:**
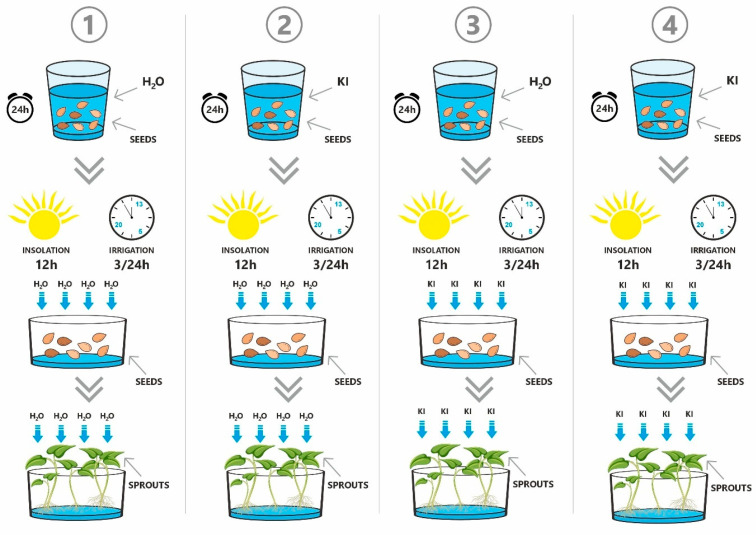
Variants of the experimental conditions applied during iodine biofortification of the *Fabaceae* sprouts (see Section 2.3 for details, 1—Variant 1; 2—Variant 2; 3—Variant 3; 4—Variant 4).

The sprouts were cultivated under four distinct experimental conditions to assess the impact of iodine biofortification (Figure 1). In Variant 1 (control, V1), the seeds were soaked for 24 h in non-carbonated spring water. The water used in the experiment had a defined composition. It was classified as moderate hardness and alkalinity water, with a calcium concentration of 51.1 mg/L and bicarbonates at 152.0 mg/L. The magnesium content was 14.4 mg/L, while chloride ions were present at 60.0 mg/L. Additionally, the water contained sodium at 18.6 mg/L, sulfates at 22.0 mg/L, and potassium at 1.31 mg/L. The fluoride concentration was low, at 0.087 mg/L, which is within the safe limits for plant growth. This water did not contain iodine. After soaking, the seeds were drained and equally distributed across five separate sprouting trays, with each plant species assigned to a separate tray. The trays were placed in an EasyGreen automatic sprouter equipped with an automated watering and aeration system featuring unidirectional water flow. The sprouts were cultivated for seven days at a controlled temperature of 23 ± 2 °C, under a 12 h light cycle and constant humidity. Watering was performed three times a day using the same water described previously in detail. In Variant 2 (V2), seeds were soaked in a 6.5 mg/L potassium iodide solution (Chempur, Piekary Śląskie, Poland), prepared using the water mentioned before, for 24 h. After soaking, the seeds were gently drained and distributed across the trays as described above. Cultivation was conducted under the same environmental and technical conditions as in Variant 1, with watering three times daily using the same water. In Variant 3 (V3), the seeds were initially soaked for 24 h in water, then drained and placed into trays. Sprouts were grown under the same controlled conditions as the previous variants but were watered three times daily with a 6.5 mg/L potassium iodide solution, prepared using the same water. In Variant 4 (V4), seeds were soaked for 24 h in the 6.5 mg/L potassium iodide solution, gently drained, and transferred to the sprouting trays.

During the 7-day cultivation period, sprouts were watered three times a day with the same iodine solution. The mean light intensity during the photoperiod (natural daylight, unshaded) was 110 ± 15 W/m^2^ (equivalent to approximately 13,200 ± 1800 lux), with the highest values observed between 11:00 and 14:00.

### 2.4. Sprout Harvesting

On the seventh day of cultivation, the sprouts were harvested, rinsed thoroughly with water, and gently dried using paper towels. The photographs of control and iodinated sprouts are presented in the Supplementary Material as Figure S1A–D. The entire sprouts (including roots, stems, and cotyledons) were homogenized using liquid nitrogen in the mortar, and approximately 1 g of homogenized plant material was weighed for analysis. The homogenized samples were then transferred into 15 mL polypropylene tubes and stored at −20 °C for further testing.

### 2.5. Biomass Rate

Before each cultivation process, the seed mass was carefully weighed. The sprouts were harvested after 7 days of cultivation and their biomass was estimated. Then, the obtained biomass of the sprouts was calculated as a % of control samples.

### 2.6. Sample Preparation and Iodine Content Evaluation

In the first step, iodine compounds were extracted from the sprouts using ultrapure water as a solvent [3]. Three portions of 1.00 ± 0.05 g of homogenized sprouts from each species were weighed and transferred into polypropylene conical tubes. Then, 3 mL of ultrapure water was added to each sample and the tubes were tightly twisted. To increase the efficiency of leaching of iodine compounds from the plant material, the contents of the tubes were subjected to ultrasounds for 10 min and then shaken for a further 15 min. The extracts were centrifuged (MPW-351R, Warszawa, Poland) for 10 min at 5000 rpm at room temperature. The resulting supernatant was poured into a new plastic vial. Due to the high concentration of iodine in the sprouts, which were grown under conditions III and IV iodine, the extractions were performed three times from the same plant material and the resulting supernatants were pooled. Thus, the complete leaching of iodine compounds from the tested sprouts was ensured. In the case of white clover sprout extract, the samples at this stage were further diluted so that the iodine concentration obtained was within the range of concentrations of the standards.

In the next step, the extracts were subjected to mineralization according to the procedure described by Machado et al. [10] with minor modifications. In brief, to 300 µL of extract was added 1 mL of 1 mol/L ammonium persulfate solution, and heated in a water bath (WB 22, MEMMERT, Schwabach, Germany) for 1 h at 95 °C. The samples were then cooled to room temperature and 2.5 mL of arsenic acid at a concentration of 0.1 mol/L was added. After 15 min, the addition of 300 µL of 80 mmol/L of ammonium cerium (IV) sulfate was started, with an interval of 30 s between samples. The solutions were mixed in a vortex shaker (V-1 plus, Biosan, Riga, Latvia) and poured into a cuvette. Exactly every 15 min from the addition of the cerium solution, the absorbance was measured using spectrophotometer Jasco V-530 (Tokyo, Japan) at a wavelength of 420 nm, corresponding to the maximum absorbance of cerium (IV). The determination was performed according to the catalytic Sandell–Kolthoff method, which is based on the reduction of Ce^4+^ to Ce^3+^ by As^3+,^ accompanied by discoloration of the yellow solution. This reaction is catalyzed by trace amounts of iodide [11]. Potassium iodate was used to prepare standard solutions of known iodine concentration (0–333 µg/L). The standard samples were subjected to the same treatments as the sprout extracts. A second-order polynomial calibration curve was fitted to the absorbance data obtained during the analysis of iodine standard solutions to describe the relationship between absorbance and iodine concentration. The coefficient of determination (R^2^ = 0.9996) indicated a strong fit of the second-order polynomial model to the experimental data. The lowest concentration of iodine solution used to prepare the calibration curve (16.7 µg/L), was established as the assay’s limit of quantification (LOQ). To confirm the stability of the analytical signal, a selected standard solution was analyzed after the measurement of each batch of 20 extract samples. The precision of sample preparation for analysis was assessed using red clover sprouts fortified with iodine according to procedures V2 and V3. For this purpose, sets of five sprouts’ samples were subjected to extraction followed by mineralization, as described above. Based on the analytical results, the relative standard deviation (RSD) was determined to be below 9%, demonstrating acceptable repeatability of the sample preparation and analysis.

### 2.7. Statistical Methods

To assess the effects of biofortification treatments (Variants V1–V4) on iodine accumulation in five legume species, we conducted a one-way ANOVA for each species independently. Post hoc comparisons were performed using NIR Fisher’s test to identify statistically significant differences between treatment groups. The normality of data distribution was checked using the Shapiro–Wilk test, and the homogeneity of variances was assessed using Levene’s test. Both assumptions were met for the analyzed datasets. These analyses were performed using STATISTICA software v. 13.3 (TIBCO Software Inc., Palo Alto, CA, USA).

## 3. Results

### 3.1. Biomass Changes

**Figure 2:**
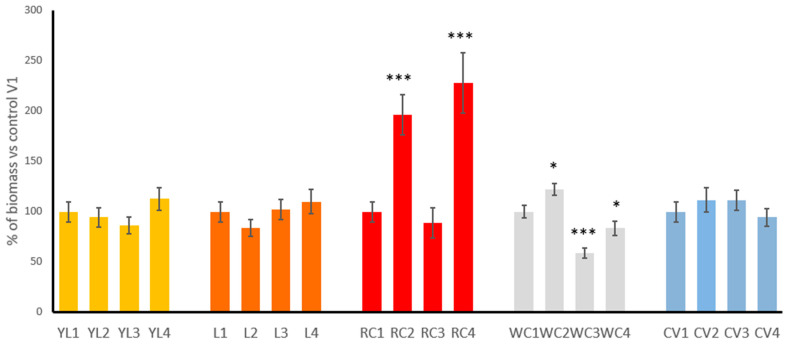
Changes in the biomass of the biofortified sprouts, presented as % of control (i.e., untreated sprouts) (YL—yellow lupine, L—lentil, RC—red clover, WC—white clover, CV—common vetch; 1–4 different variants of biofortification procedure, see Section 2.3 for details). Asterisks indicate statistically significant differences compared to the respective control group: *p* < 0.05 (*), *p* < 0.001 (***).

Figure 2 illustrates the relative biomass of the sprouts of five legume species cultivated under four different experimental variants (V1–V4), expressed as a percentage of the control condition (V1) for each plant. The results demonstrate how each species responded to iodine biofortification strategies.

In yellow lupine, lentil and common vetch sprouts, biomass remained relatively stable across all treatment variants, indicating that iodine treatments had little effect—either positive or negative—on biomass in these species. In case of clover species, the most visible influence of iodination was observed. Red clover responded exceptionally well to iodine biofortification. While the control group (RC1) maintained baseline biomass, RC2 sprouts showed a strong increase to about 200%, and RC4 sprouts exhibited the highest overall biomass, reaching nearly 250% of the control. These increases are statistically significant (*p* < 0.001) and indicate that red clover not only tolerates iodine well but also benefits from it, particularly when both soaking and watering methods are used together (V4). Conversely, the RC3 group showed a slight decline, suggesting that watering alone may be less effective than soaking or combining both methods as it primarily affects the soil and may result in reduced iodine availability for developing sprouts due to adsorption, leaching, or uneven distribution of the solution. In white clover, the biomass response was more variable. WC2 sprouts showed a moderate increase *(p* < 0.05) in biomass (around 130–140%), whereas WC3 sprouts exhibited a substantial decline to approximately 60%, indicating a statistically significant reduction (*p* < 0.001). This suggests that watering alone with iodine (V3) may have a phytotoxic effect on this species. The biomass of WC4 sprouts was reduced to that of the control *(p* < 0.05), while WC2 sprouts showed a positive effect, pointing to soaking (V2) as a potentially beneficial method for this plant.

### 3.2. Iodine Concentration in Sprouts Under Different Biofortification Treatments

The results of iodine incorporation into the sprouts grown in different biofortification patterns and conversion to percentage of the recommended dietary allowance for this trace element in adults are presented in Table 1 and Table 2, respectively.

The sprouts from the control group (V1) showed minimal or non-detectable iodine levels across all species, confirming that the baseline iodine content in the untreated sprouts is negligible. The sprouts obtained by prior soaking the seeds in iodine solution (V2) resulted in low but detectable iodine accumulation, with statistically significant increases compared to V1 in all examined species (except RC and WC, where the differences were not always significant). Watering sprouts with iodine solution (V3) revealed the highest iodine concentrations in the sprouts of all tested species, indicating this treatment as the most effective single-method strategy. Variant 4 (soaking + watering, both with iodine solution) showed slightly lower iodine content than V3 in most species, suggesting a potential saturation point or a regulatory mechanism limiting further iodine uptake when the exposure is extended.

White clover showed the highest iodine accumulation (1026.7 ± 60.4 µg I/100 g fw in V3), making it the most efficient iodine accumulator under the given conditions but at the same time carrying the risk of excessive iodine intake (684% RDA) for the potential consumer. Common vetch and lentil also demonstrated high uptake, suggesting they are promising candidates for biofortification programs.

In Experiment V2, yellow lupine accumulated the highest iodine levels (10.4 ± 1.7 µg/100 g fw), significantly higher than those observed in red and white clover, and common vetch (*p* < 0.01). In contrast, lentil accumulated the lowest iodine content (2.8 ± 1.8 µg/100 g fw), which was significantly lower compared to all other tested species (*p* < 0.001). These results clearly indicate a species-dependent capacity for iodine bioaccumulation under the soaking (V2) biofortification method. In Experiment V3, white clover showed by far the highest iodine accumulation (1026.7 ± 60.4 µg/100 g fw), significantly exceeding all other species (*p* < 0.001). Common vetch and red clover also exhibited relatively high iodine contents, whereas lentil and yellow lupine accumulated significantly lower amounts (*p* < 0.01). These findings further confirm a distinct, species-specific pattern of iodine uptake under the watering (V3) treatment. The notably higher iodine content in white clover sprouts suggests an enhanced ability to absorb and retain iodine, while the relatively low levels in yellow lupine and lentil point to physiological constraints that may limit iodine uptake in these species. In Experiment V4, white clover again exhibited the highest iodine accumulation, significantly surpassing all other species (*p* < 0.001). Moderate levels were recorded in common vetch, red clover, and yellow lupine, with no statistically significant differences among these species. The lowest iodine content was again observed in lentil sprouts, significantly lower than in white clover and common vetch (*p* < 0.01). These results demonstrate a strong species-dependent efficiency of iodine biofortification via the combined soaking and watering method (V4). In particular, white clover consistently showed superior uptake ability, while lentil remained the least efficient accumulator under these conditions.

## 4. Discussion

Biofortification of sprouts with iodine is problematic, because not all plants are able to accumulate this trace element. It is linked to the fact that iodine is not considered essential for land plants [12]. In general, iodate has more favorable effect on growth than iodide, particularly at the initial stages of the development. What should be noted, excessive iodine has an evident inhibitory and even toxic effect on plant growth, as it can accelerate the oxidation of plant tissues and senescence [6,13]. The most common chemical compounds used in fortification of the sprouts were inorganic salts, like potassium iodide, potassium iodate [4]. Iodine biofortification of leafy vegetables was obtained so far for spinach, lettuce, celery, and different kinds of cabbages. We did not identify any study using legume plants. Until now only a few examples of iodinated sprouts were reported (e.g., buckwheat, kohlrabi, pumpkin) [14,15,16].

Osmić et al., (2017) investigated the effects of selenium and iodine—applied in the forms of iodide and iodate—on kohlrabi sprouts. The chemical form of iodine significantly influenced pigment composition, plant morphology, and photosystem II efficiency, though it did not have a notable impact on germination [4]. Research on buckwheat and pumpkin sprouts further demonstrated that different forms of iodine and selenium affected pigment levels and photosynthetic performance, underlining the importance of the chemical form in biofortification results [13]. Golob et al. (2020) [17] explored selenium and iodine co-fortification in pumpkin sprouts and reported enhanced iodine accumulation, along with interactive effects between the two trace elements that influenced seed germination and sprout biomass [17]. Similarly, Jerše et al. (2017) found that pea seeds treated with iodine and selenium solutions exhibited increased accumulation of both elements and notable changes in morphological and physiological traits [6]. In our study, iodine-biofortified seedlings developed at a rate comparable to the control group and showed no signs of phytotoxicity such as chlorosis, necrosis, or other visible stress symptoms caused by iodination. This indicates that the potassium iodide concentrations applied were well tolerated. The effectiveness of iodine enrichment has also been confirmed in other crops, including spinach [18], lettuce [19,20], tomatoes and potatoes [21], carrots [22], and radishes [23]. In many of these species, the accumulation of iodine depends on xylem transport, which can limit the concentration achieved in edible parts [24]. However, this limitation is irrelevant for sprouts, as the whole plant is consumed.

Previous research suggests that iodine applied in the form of iodide generally results in higher accumulation compared to iodate, as seen in lettuce, tomatoes, and potatoes. Although iodide is more readily absorbed by plants, it is also more phytotoxic than iodate [24,25]. Since plants naturally reduce iodate to iodide before uptake, applying iodide directly—while carefully controlling its concentration—can be an efficient and effective approach to biofortification. In our view, this may indicate that iodide is a valuable agent for enhancing the iodine content of edible plants like sprouts, but it needs to be confirmed in future studies.

Food fortified with iodine seems to be crucial for people avoiding animal-derived food (i.e., vegetarians and vegans), thus biofortification of sprouts could be a good and healthy alternative source of iodine. But the range of amount of iodine in such products should be strictly controlled, especially in case of the subjects with abnormal thyroid function. In the process of fortification with trace elements like iodine, not only their total concentration in the sprouts/plants should be evaluated, but also the chemical speciation. Until now, this kind of study has never been recognized. It is crucial due to different iodine derivates, such as iodolactones and iodohexadecanal, which are noted in the plant after iodine treatment.

The assessment of iodine absorption capacity in the seeds and sprouts of the selected plant species, as well as the determination of an optimal iodine dose, formed the core objective of this research stage. Broader studies aimed at identifying and comparing various iodine forms and compounds are planned for the future. It is generally assumed that iodide (I^−^) is the predominant form of iodine present in plant tissues [26]. However, organic iodine compounds, such as iodosalicylates and iodobenzoates, have also been explored in plant biofortification [27]. Krzepiłko et al. (2021) demonstrated that a single application of potassium iodide at the concentrations ranging from 0.15 to 1.50 mg/g of seeds effectively enhanced iodine content in radish sprouts via seed germination. The tested radish cultivars exhibited strong iodine uptake and produced high-quality seedlings [24].

In our earlier analyses, the natural iodine content in lentil sprouts had been determined at a modest level of 0.07 ± 0.02 mg/100 g of dry matter [3]. In contrast, the current study revealed a substantially higher average concentration, reaching 0.7 ± 0.3 mg/100 g when expressed on a fresh weight basis. Considering that lentil sprouts typically contain approximately 90% water, the dry matter and fresh weight calculations are in fact consistent, and this agreement provides further confirmation of the robustness and reliability of the analytical methods employed in both investigations. Beyond the methodological validation, these findings underscore the broader nutritional relevance of sprouted legumes. Sprouts from pulses such as lentils, peas, or beans are not only reservoirs of valuable trace and essential elements but also represent an exceptionally rich source of plant-derived proteins of high biological value. This dual nutritional potential becomes particularly important in the context of dietary trends observed in recent years, with an increasing proportion of the global population turning to plant-based or fully vegetarian diets [1]. For the individuals excluding animal products, legumes and their sprouts can play a pivotal role in securing adequate protein intake, while at the same time addressing deficiencies in essential micronutrients such as iodine, which is often limited in plant-based diets. For example, approximately 15 g of white clover sprouts from variant V3 could supply 100% RDA for iodine in the adult population, which additionally highlights the feasibility of incorporating this amount of it into daily diet. On the other hand, however, it is important to remember about the risks associated with excessive iodine intake, especially when such sprouts would form the basis of a meal rather than just one of its components. The possibility of combining these two nutritional benefits—valuable protein provision and micronutrient fortification—makes the sprouted legumes an especially attractive dietary component. Sprouts enriched with iodine, whether naturally or through targeted biofortification strategies, may serve as a functional food capable of supplying this critical trace element alongside high-quality plant protein. This is of particular significance in vegetarian and vegan diets, where the lack of marine foods (a primary natural source of iodine) often predisposes individuals to suboptimal iodine status. Lentil sprouts, in particular, appear to offer an ideal balance, combining high acceptability, short germination time, excellent digestibility, and the ability to accumulate meaningful amounts of iodine.

## 5. Conclusions

Of all the tested cultivation conditions, the variant in which seeds were initially soaked in water for 24 h and the sprouts were subsequently watered three times daily with a 6.5 mg/L potassium iodide solution appeared to be the most optimal for the iodine biofortification of all tested legume species. Among the plants examined, white clover was the most efficient in accumulating iodine, followed by common vetch and lentils. These findings support the potential of using sprouts as functional foods enriched with iodine, particularly in vegetarian or iodine-deficient diets. Red clover treated with combining seed soaking and watering achieved the highest biomass overall, making it the most promising combination of species and treatment method. Among all the iodine application strategies, this method proved to be the most effective and consistent in enhancing biomass across multiple species without inducing phytotoxic effects.

Our study highlights how potassium iodide affects sprouting dynamics, providing insights into optimizing germination processes for iodine fortification. These studies emphasize the promising potential of iodine biofortification for improving both nutritional content and growth parameters in sprouting legume plants. Therefore, our current and previous findings not only expand the scientific understanding of the nutritional profiles of legume sprouts but also highlight their strategic importance for public health nutrition. The dual role of legume sprouts—as the carriers of essential minerals and as rich sources of valuable plant protein—underlines their unique capacity to address multiple nutritional needs simultaneously. Nevertheless, it is important to note that the chemical form of iodine plays a key role in determining the effectiveness and safety of biofortification strategies. The lack of analysis of the chemical form of iodine and its bioavailability are undoubtedly a limitation of our pilot study and should be thoroughly assessed in the future, similar to iodine stability evaluation during sprout storage.

The possibility of fortifying sprouts with iodine provides an innovative approach to counteracting dietary deficiencies in this essential micronutrient, while at the same time offering a sustainable, plant-based protein source. This synergy ensures that sprouted legumes could be an easily accessible part of the daily diet, increasing the supply of protein and iodine, which can be particularly important for populations following a plant-based diet, as well as for broader global strategies aimed at sustainable nutrition.

## Figures and Tables

**Table 1:** Total iodine concentration in the sprouts under different biofortification treatments (µg I/100 g fresh weight).

Concentration of Iodine [μg I/100 g fw]	V1	V2	V3	V4
yellow lupine (YL) sprouts	<LOQ ^1^	10.4 ± 1.7 ^a^	157.3 ± 4.5 ^b^	217.7 ± 5.5 ^c^
red clover (RC) sprouts	<LOQ ^1^	7.0 ± 1.2 ^a^	208.7 ± 6.4 ^b^	230.7 ± 9.8 ^b^
lentil (L) sprouts	07 ± 0.3 ^a^	2.8 ± 1.8 ^a^	201.5 ± 18.1 ^b^	160.7 ± 5.4 ^b^
white clover (WC) sprouts	1.1 ± 0.1 ^a^	5.1 ± 1.5 ^a^	1026.7 ± 60.4 ^b^	857.1 ± 30.5 ^b^
common vetch (CV) sprouts	<LOQ ^1^	5.7 ± 1.2 ^a^	266.8 ± 3.8 ^b^	238.7 ± 8.9 ^b^

^1^ LOQ—limit of quantification; values with different letters (a, b, c) are significantly different at *p* < 0.05 within rows; V1–V4 different variants of biofortification procedure (see Section 2.3 for details).

**Table 2:** Fulfillment of the recommended daily allowance for iodine by consumption of 100 g of fresh examined sprouts (% RDA * I/100 g of fresh sprouts).

Fulfillment of the Recommended Daily Allowance for Iodine [% RDA */100 g Fresh Sprouts]	V1	V2	V3	V4
yellow lupine (YL) sprouts	-	7	105	145
red clover (RC) sprouts	-	5	139	154
lentil (L) sprouts	<1	2	134	107
white clover (WC) sprouts	<1	3	684	571
common vetch (CV) sprouts	-	4	178	159

* RDA for adult is 150 µg.

## Data Availability

Data are contained within the article, further inquiries can be directed to the corresponding author.

## Supplementary Materials

The following supporting information can be downloaded at: https://www.mdpi.com/article/10.3390/foods14213691/s1. Figure S1A. Sprouts harvested in Variant 1 (control, V1). Figure S1B. Sprouts harvested in Variant 2 (V2). Figure S1C. Sprouts harvested in Variant 3 (V3). Figure S1D. Sprouts harvested in Variant 4 (V4).
