# Supplementary material for: Iodine Fortification of Edible Legume Sprouts: A Pilot Biofortification Study
Source: Foods. 2025 Oct 29;14(21):3691. doi: 10.3390/foods14213691 (PMC12610263; doi:10.3390/foods14213691)
Supplement: Supplementary file 1 [file foods-14-03691-s001.zip › foods-3865142-supplementary.pdf]

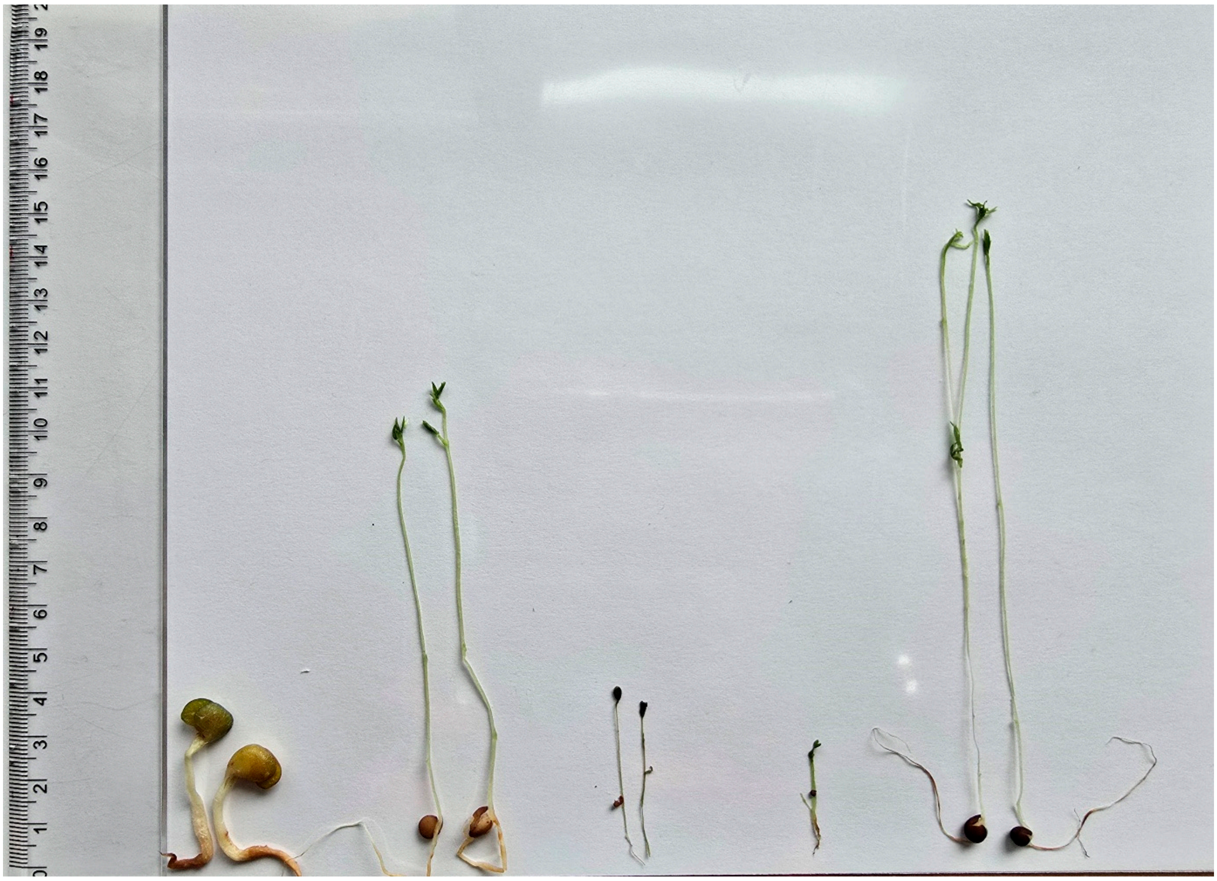

Figure S1A. Sprouts harvested in Variant 1 (control, V1). Growth conditions: seeds were soaked for 24 hours in non-carbonated spring water, then gently drained and transferred to sprouting trays. Sprouts were cultivated under controlled environmental conditions identical for all experimental variants. The photograph presents two representative examples for each species, shown in the following order: YL – yellow lupine, L – lentil, RC – red clover, WC – white clover, and CV – common vetch.

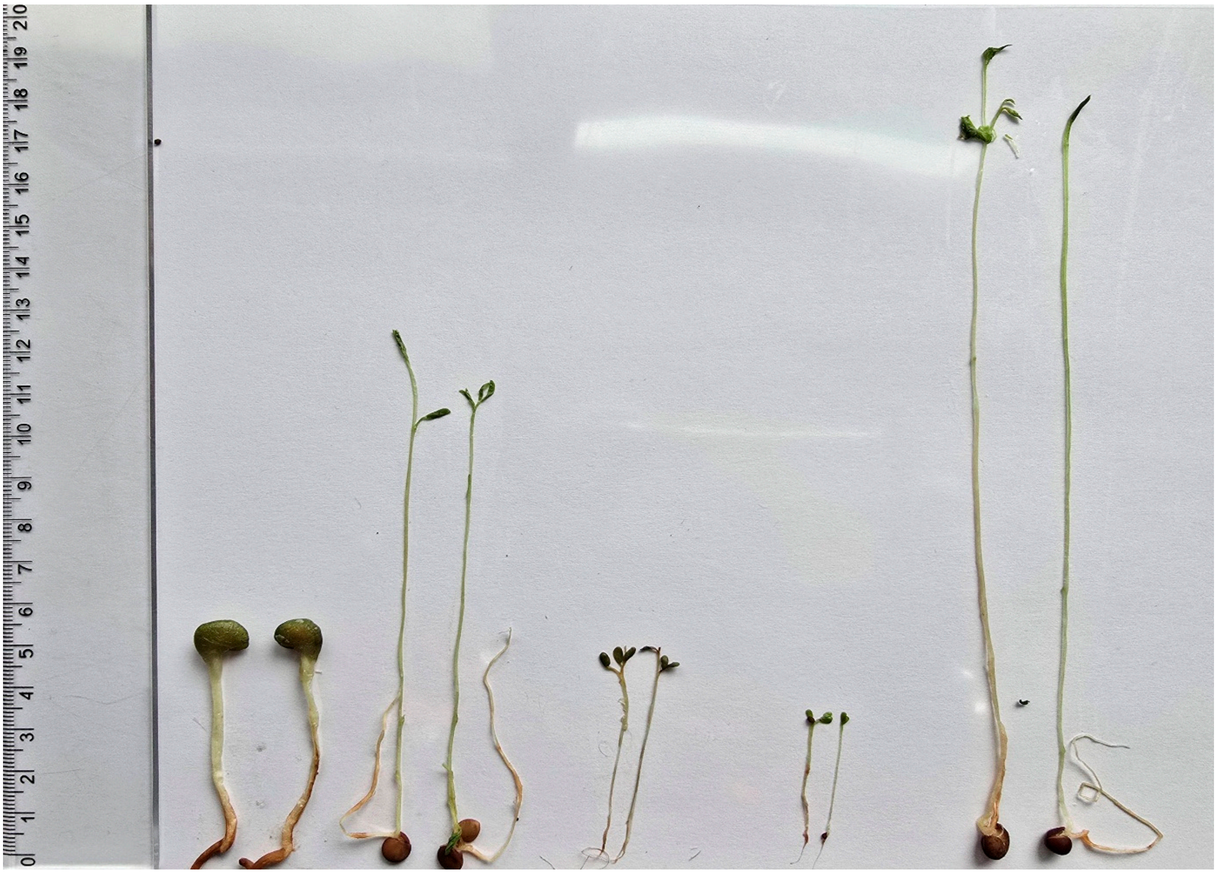

Figure S1B. Sprouts harvested in Variant 2 (V2). Growth conditions: seeds were soaked for 24 hours in a 6.5 mg/L potassium iodide (KI) solution prepared with the same water as used in the control variant. After soaking, the seeds were gently drained and transferred to sprouting trays, then cultivated under the same controlled conditions as in Variant 1 (V1). The photograph presents two representative examples for each species, shown in the following order: YL – yellow lupine, L – lentil, RC – red clover, WC – white clover, and CV – common vetch.

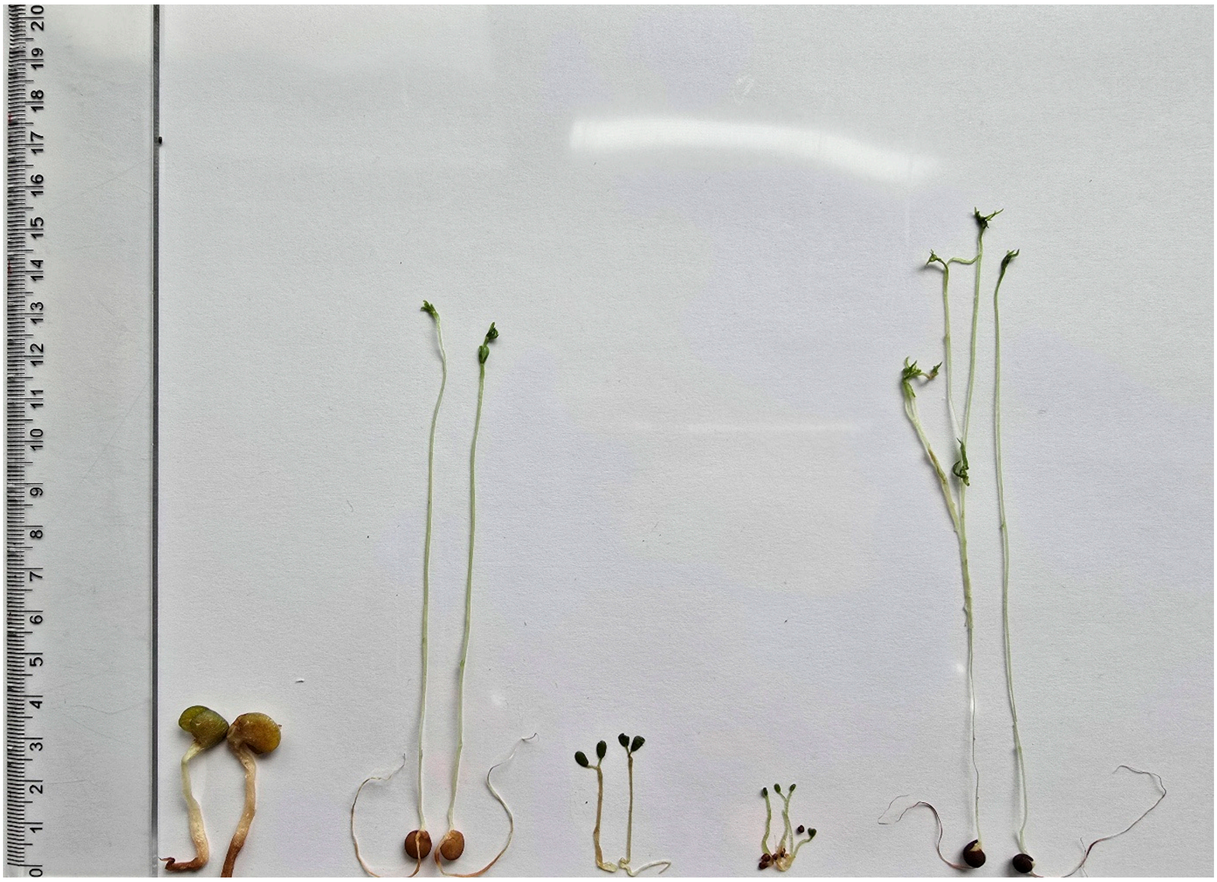

Figure S1C. Sprouts harvested in Variant 3 (V3). Growth conditions: seeds were soaked in water for 24 hours, then drained and placed into trays. Sprouts were grown under the same controlled conditions as in Variants 1 (V1) and 2 (V2), but were watered three times daily with a 6.5 mg/L potassium iodide (KI) solution. The photograph presents two representative examples for each species, shown in the following order: YL – yellow lupine, L – lentil, RC – red clover, WC – white clover, and CV – common vetch.

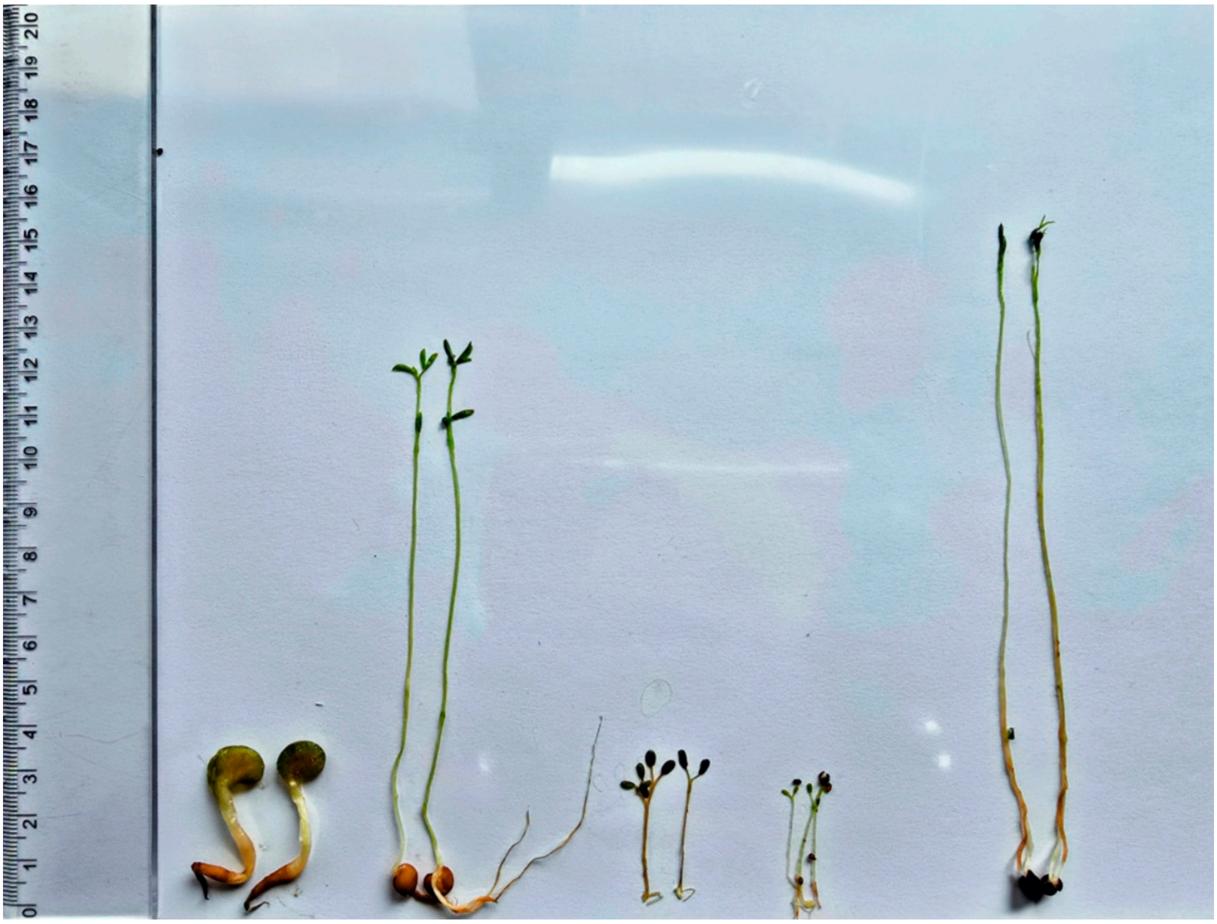

Figure S1D. Sprouts harvested in Variant 4 (V4). Growth conditions: seeds were soaked for 24 hours in a 6.5 mg/L potassium iodide (KI) solution, gently drained, and transferred to sprouting trays. During cultivation, the sprouts were watered three times daily with the same KI solution under controlled conditions. The photograph presents two representative examples for each species, shown in the following order: YL – yellow lupine, L – lentil, RC – red clover, WC – white clover, and CV – common vetch.
